# Supplementary material for: Role and mechanism of NCAPD3 in promoting malignant behaviors in gastric cancer
Source: Front Pharmacol. 2024 Apr 22;15:1341039. doi: 10.3389/fphar.2024.1341039 (PMC11070777; doi:10.3389/fphar.2024.1341039)
Supplement: Supplementary file 11 [file DataSheet2.ZIP › GSEA/Transcipt factor/my_analysis.Gsea.1599461814734/THAP1_TARGET_GENES.html]

Details for gene set THAP1\_TARGET\_GENES[GSEA]

|  || Dataset | filtered\_dataset.sample\_info.cls#WT\_versus\_NCAPD3\_MUT |
| Phenotype | sample\_info.cls#WT\_versus\_NCAPD3\_MUT |
| Upregulated in class | WT |
| GeneSet | THAP1\_TARGET\_GENES |
| Enrichment Score (ES) | 0.24473457 |
| Normalized Enrichment Score (NES) | 1.8857431 |
| Nominal p-value | 0.0015174507 |
| FDR q-value | 0.2647728 |
| FWER p-Value | 0.904 |
Table: GSEA Results Summary

  

Fig 1: Enrichment plot: THAP1\_TARGET\_GENES      
 Profile of the Running ES Score & Positions of GeneSet Members on the Rank Ordered List

  

| SYMBOL | TITLE | RANK IN GENE LIST | RANK METRIC SCORE | RUNNING ES | CORE ENRICHMENT || 1 | 51111 | KMT5B | 2 | 1.217 | 0.0128 | Yes |
| 2 | 29070 | CCDC113 | 8 | 1.114 | 0.0221 | Yes |
| 3 | 9759 | HDAC4 | 46 | 0.925 | 0.0035 | Yes |
| 4 | 1497 | CTNS | 58 | 0.883 | 0.0052 | Yes |
| 5 | 7743 | ZNF189 | 74 | 0.851 | 0.0033 | Yes |
| 6 | 153364 | MBLAC2 | 79 | 0.838 | 0.0100 | Yes |
| 7 | 51540 | SCLY | 95 | 0.800 | 0.0075 | Yes |
| 8 | 79020 | C7orf25 | 97 | 0.793 | 0.0161 | Yes |
| 9 | 55006 | TRMT61B | 101 | 0.782 | 0.0230 | Yes |
| 10 | 7769 | ZNF226 | 125 | 0.777 | 0.0138 | Yes |
| 11 | 55687 | TRMU | 128 | 0.774 | 0.0214 | Yes |
| 12 | 286101 | ZNF252P | 136 | 0.758 | 0.0248 | Yes |
| 13 | 339318 | ZNF181 | 146 | 0.740 | 0.0264 | Yes |
| 14 | 10929 | SRSF8 | 150 | 0.735 | 0.0328 | Yes |
| 15 | 22879 | MON1B | 151 | 0.735 | 0.0415 | Yes |
| 16 | 51174 | TUBD1 | 153 | 0.730 | 0.0494 | Yes |
| 17 | 7733 | ZNF180 | 154 | 0.728 | 0.0580 | Yes |
| 18 | 2186 | BPTF | 159 | 0.717 | 0.0633 | Yes |
| 19 | 54971 | BANP | 172 | 0.707 | 0.0621 | Yes |
| 20 | 57325 | CSRP2BP | 176 | 0.698 | 0.0680 | Yes |
| 21 | 84190 | METTL25 | 195 | 0.684 | 0.0617 | Yes |
| 22 | 9897 | KIAA0196 | 197 | 0.682 | 0.0690 | Yes |
| 23 | 145389 | SLC38A6 | 202 | 0.673 | 0.0738 | Yes |
| 24 | 126792 | B3GALT6 | 223 | 0.658 | 0.0656 | Yes |
| 25 | 84816 | RTN4IP1 | 232 | 0.649 | 0.0670 | Yes |
| 26 | 57521 | RPTOR | 239 | 0.645 | 0.0698 | Yes |
| 27 | 55238 | SLC38A7 | 250 | 0.630 | 0.0693 | Yes |
| 28 | 8498 | RANBP3 | 251 | 0.629 | 0.0768 | Yes |
| 29 | 528 | ATP6V1C1 | 254 | 0.625 | 0.0826 | Yes |
| 30 | 25973 | PARS2 | 266 | 0.620 | 0.0812 | Yes |
| 31 | 92595 | ZNF764 | 273 | 0.616 | 0.0837 | Yes |
| 32 | 80124 | VCPIP1 | 282 | 0.607 | 0.0845 | Yes |
| 33 | 84874 | ZNF514 | 283 | 0.606 | 0.0917 | Yes |
| 34 | 23036 | ZNF292 | 288 | 0.602 | 0.0956 | Yes |
| 35 | 6715 | SRD5A1 | 325 | 0.578 | 0.0737 | Yes |
| 36 | 8125 | ANP32A | 326 | 0.578 | 0.0806 | Yes |
| 37 | 7220 | TRPC1 | 328 | 0.576 | 0.0866 | Yes |
| 38 | 94107 | TMEM203 | 333 | 0.574 | 0.0902 | Yes |
| 39 | 4297 | KMT2A | 337 | 0.573 | 0.0946 | Yes |
| 40 | 2342 | FNTB | 344 | 0.570 | 0.0966 | Yes |
| 41 | 92399 | MRRF | 347 | 0.569 | 0.1018 | Yes |
| 42 | 2965 | GTF2H1 | 356 | 0.563 | 0.1020 | Yes |
| 43 | 5810 | RAD1 | 360 | 0.560 | 0.1063 | Yes |
| 44 | 10818 | FRS2 | 363 | 0.558 | 0.1113 | Yes |
| 45 | 5036 | PA2G4 | 365 | 0.557 | 0.1171 | Yes |
| 46 | 115509 | ZNF689 | 374 | 0.551 | 0.1173 | Yes |
| 47 | 10212 | DDX39A | 389 | 0.543 | 0.1125 | Yes |
| 48 | 5930 | RBBP6 | 390 | 0.543 | 0.1190 | Yes |
| 49 | 9815 | GIT2 | 394 | 0.540 | 0.1230 | Yes |
| 50 | 5814 | PURB | 399 | 0.536 | 0.1262 | Yes |
| 51 | 60526 | LDAH | 403 | 0.535 | 0.1301 | Yes |
| 52 | 147007 | TMEM199 | 410 | 0.533 | 0.1316 | Yes |
| 53 | 54994 | GID8 | 415 | 0.526 | 0.1347 | Yes |
| 54 | 10885 | WDR3 | 430 | 0.515 | 0.1296 | Yes |
| 55 | 148304 | C1orf74 | 447 | 0.506 | 0.1228 | Yes |
| 56 | 57478 | USP31 | 448 | 0.506 | 0.1288 | Yes |
| 57 | 54482 | TRMT13 | 449 | 0.506 | 0.1348 | Yes |
| 58 | 8607 | RUVBL1 | 451 | 0.506 | 0.1400 | Yes |
| 59 | 200933 | FBXO45 | 456 | 0.500 | 0.1428 | Yes |
| 60 | 29071 | C1GALT1C1 | 457 | 0.500 | 0.1487 | Yes |
| 61 | 27097 | TAF5L | 461 | 0.498 | 0.1522 | Yes |
| 62 | 7109 | TRAPPC10 | 475 | 0.488 | 0.1476 | Yes |
| 63 | 140775 | SMCR8 | 487 | 0.480 | 0.1445 | Yes |
| 64 | 29101 | SSU72 | 496 | 0.475 | 0.1438 | Yes |
| 65 | 11060 | WWP2 | 503 | 0.471 | 0.1446 | Yes |
| 66 | 5188 | GATB | 512 | 0.469 | 0.1438 | Yes |
| 67 | 104 | ADARB1 | 518 | 0.463 | 0.1452 | Yes |
| 68 | 57180 | ACTR3B | 526 | 0.460 | 0.1451 | Yes |
| 69 | 90639 | COX19 | 535 | 0.454 | 0.1441 | Yes |
| 70 | 29090 | TIMM21 | 537 | 0.453 | 0.1487 | Yes |
| 71 | 8195 | MKKS | 542 | 0.449 | 0.1508 | Yes |
| 72 | 3609 | ILF3 | 544 | 0.449 | 0.1554 | Yes |
| 73 | 9416 | DDX23 | 553 | 0.445 | 0.1542 | Yes |
| 74 | 79080 | CCDC86 | 555 | 0.443 | 0.1587 | Yes |
| 75 | 78988 | MRPL57 | 557 | 0.441 | 0.1632 | Yes |
| 76 | 147015 | DHRS13 | 559 | 0.441 | 0.1676 | Yes |
| 77 | 53615 | MBD3 | 561 | 0.441 | 0.1720 | Yes |
| 78 | 7112 | TMPO | 566 | 0.439 | 0.1740 | Yes |
| 79 | 84335 | AKT1S1 | 572 | 0.437 | 0.1752 | Yes |
| 80 | 27349 | MCAT | 574 | 0.436 | 0.1796 | Yes |
| 81 | 79053 | ALG8 | 576 | 0.436 | 0.1840 | Yes |
| 82 | 55245 | UQCC1 | 580 | 0.434 | 0.1868 | Yes |
| 83 | 9816 | URB2 | 581 | 0.434 | 0.1919 | Yes |
| 84 | 90806 | ANGEL2 | 583 | 0.434 | 0.1963 | Yes |
| 85 | 26164 | MTG2 | 585 | 0.433 | 0.2006 | Yes |
| 86 | 23195 | MDN1 | 596 | 0.426 | 0.1977 | Yes |
| 87 | 55039 | TRMT12 | 599 | 0.424 | 0.2011 | Yes |
| 88 | 6208 | RPS14 | 604 | 0.421 | 0.2029 | Yes |
| 89 | 23404 | EXOSC2 | 609 | 0.418 | 0.2047 | Yes |
| 90 | 8021 | NUP214 | 611 | 0.417 | 0.2088 | Yes |
| 91 | 7290 | HIRA | 612 | 0.417 | 0.2138 | Yes |
| 92 | 56339 | METTL3 | 613 | 0.417 | 0.2187 | Yes |
| 93 | 55308 | DDX19A | 615 | 0.416 | 0.2229 | Yes |
| 94 | 22847 | ZNF507 | 618 | 0.413 | 0.2262 | Yes |
| 95 | 54663 | WDR74 | 628 | 0.408 | 0.2238 | Yes |
| 96 | 11083 | DIDO1 | 631 | 0.406 | 0.2270 | Yes |
| 97 | 10274 | STAG1 | 633 | 0.406 | 0.2311 | Yes |
| 98 | 9852 | EPM2AIP1 | 634 | 0.406 | 0.2359 | Yes |
| 99 | 29890 | RBM15B | 651 | 0.400 | 0.2278 | Yes |
| 100 | 23250 | ATP11A | 664 | 0.395 | 0.2229 | Yes |
| 101 | 51603 | METTL13 | 669 | 0.391 | 0.2244 | Yes |
| 102 | 5134 | PDCD2 | 678 | 0.388 | 0.2226 | Yes |
| 103 | 157570 | ESCO2 | 685 | 0.386 | 0.2224 | Yes |
| 104 | 55157 | DARS2 | 686 | 0.385 | 0.2269 | Yes |
| 105 | 28996 | HIPK2 | 687 | 0.385 | 0.2315 | Yes |
| 106 | 10452 | TOMM40 | 696 | 0.380 | 0.2296 | Yes |
| 107 | 10607 | TBL3 | 698 | 0.380 | 0.2333 | Yes |
| 108 | 84975 | MFSD5 | 702 | 0.376 | 0.2354 | Yes |
| 109 | 23076 | RRP1B | 709 | 0.370 | 0.2350 | Yes |
| 110 | 84172 | POLR1B | 718 | 0.365 | 0.2329 | Yes |
| 111 | 23350 | U2SURP | 719 | 0.364 | 0.2372 | Yes |
| 112 | 93487 | MAPK1IP1L | 723 | 0.362 | 0.2391 | Yes |
| 113 | 9131 | AIFM1 | 728 | 0.360 | 0.2402 | Yes |
| 114 | 134430 | WDR36 | 733 | 0.359 | 0.2413 | Yes |
| 115 | 23640 | HSPBP1 | 735 | 0.359 | 0.2447 | Yes |
| 116 | 8772 | FADD | 742 | 0.355 | 0.2441 | No |
| 117 | 58509 | CACTIN | 748 | 0.351 | 0.2443 | No |
| 118 | 10726 | NUDC | 754 | 0.346 | 0.2444 | No |
| 119 | 55646 | LYAR | 761 | 0.342 | 0.2437 | No |
| 120 | 9238 | TBRG4 | 786 | 0.330 | 0.2284 | No |
| 121 | 4285 | MIPEP | 815 | 0.307 | 0.2097 | No |
| 122 | 9221 | NOLC1 | 816 | 0.307 | 0.2133 | No |
| 123 | 9343 | EFTUD2 | 818 | 0.305 | 0.2161 | No |
| 124 | 387680 | FAM21A | 828 | 0.297 | 0.2125 | No |
| 125 | 253725 | FAM21C | 829 | 0.297 | 0.2160 | No |
| 126 | 3146 | HMGB1 | 831 | 0.295 | 0.2187 | No |
| 127 | 1984 | EIF5A | 846 | 0.259 | 0.2106 | No |
| 128 | 3312 | HSPA8 | 848 | 0.244 | 0.2127 | No |
| 129 | 50640 | PNPLA8 | 881 | -0.296 | 0.1906 | No |
| 130 | 571 | BACH1 | 884 | -0.300 | 0.1926 | No |
| 131 | 27131 | SNX5 | 907 | -0.333 | 0.1789 | No |
| 132 | 8738 | CRADD | 922 | -0.349 | 0.1719 | No |
| 133 | 8780 | RIOK3 | 948 | -0.367 | 0.1562 | No |
| 134 | 80336 | PABPC1L | 971 | -0.387 | 0.1432 | No |
| 135 | 11077 | HSF2BP | 1008 | -0.408 | 0.1193 | No |
| 136 | 1628 | DBP | 1029 | -0.429 | 0.1084 | No |
| 137 | 7871 | SLMAP | 1038 | -0.435 | 0.1071 | No |
| 138 | 10616 | RBCK1 | 1042 | -0.438 | 0.1099 | No |
| 139 | 80315 | CPEB4 | 1053 | -0.448 | 0.1073 | No |
| 140 | 81631 | MAP1LC3B | 1060 | -0.452 | 0.1078 | No |
| 141 | 196394 | AMN1 | 1079 | -0.461 | 0.0989 | No |
| 142 | 100534599 | ISY1-RAB43 | 1082 | -0.462 | 0.1028 | No |
| 143 | 3669 | ISG20 | 1097 | -0.475 | 0.0972 | No |
| 144 | 2729 | GCLC | 1123 | -0.497 | 0.0831 | No |
| 145 | 8444 | DYRK3 | 1131 | -0.502 | 0.0835 | No |
| 146 | 146057 | TTBK2 | 1148 | -0.513 | 0.0768 | No |
| 147 | 7518 | XRCC4 | 1175 | -0.540 | 0.0624 | No |
| 148 | 57649 | PHF12 | 1212 | -0.588 | 0.0406 | No |
| 149 | 29968 | PSAT1 | 1215 | -0.589 | 0.0460 | No |
| 150 | 56995 | TULP4 | 1216 | -0.591 | 0.0530 | No |
| 151 | 4189 | DNAJB9 | 1223 | -0.598 | 0.0553 | No |
| 152 | 22931 | RAB18 | 1231 | -0.608 | 0.0569 | No |
| 153 | 440 | ASNS | 1232 | -0.609 | 0.0642 | No |
| 154 | 10802 | SEC24A | 1251 | -0.639 | 0.0574 | No |
| 155 | 8412 | BCAR3 | 1261 | -0.648 | 0.0579 | No |
| 156 | 83931 | STK40 | 1281 | -0.669 | 0.0506 | No |
| 157 | 57533 | TBC1D14 | 1304 | -0.708 | 0.0414 | No |
| 158 | 23365 | ARHGEF12 | 1329 | -0.751 | 0.0311 | No |
| 159 | 7706 | TRIM25 | 1357 | -0.817 | 0.0192 | No |
| 160 | 23431 | AP4E1 | 1359 | -0.827 | 0.0283 | No |
| 161 | 2040 | STOM | 1389 | -0.988 | 0.0168 | No |
Table: GSEA details [plain text format]

  

Fig 2: THAP1\_TARGET\_GENES      
 Blue-Pink O' Gram in the Space of the Analyzed GeneSet

  

Fig 3: THAP1\_TARGET\_GENES: Random ES distribution      
 Gene set null distribution of ES for **THAP1\_TARGET\_GENES**
